# New Horizons in Nanofiller-Based Polymer Composites II

**DOI:** 10.3390/polym15214259

**Published:** 2023-10-30

**Authors:** Vineet Kumar, Xiaowu Tang

**Affiliations:** 1 School of Mechanical Engineering, Yeungnam University, 280 Daehak-Ro, Gyeongsan 38541, Republic of Korea; 2 College of Material and Chemical Engineering, Zhengzhou University of Light Industry, Zhengzhou 450001, China; tangxiaowu@naver.com

## 1. Introduction

Nanofiller-based polymer composites are a hot-topic research area with significant industrial potential. These polymer composites are reinforced with different classes of filler with at least one dimension and a grain size below 100 nm. These nanoscale fillers are carbon-based, such as carbon nanotubes, graphene, and nano-carbon black [1]. Other nanofiller types include silica and clay minerals. Among these nanofillers, carbon nanotubes and graphene in particular have been extensively explored for their potential to improve mechanical, electrical, and thermal properties. Moreover, clay minerals have been studied for their ability to improve reinforcing properties or barrier properties. Besides nanofillers, polymer matrices are effective for obtaining robust composites [2]. These polymers can be thermoplastic, thermosets, or elastomers. Among them, elastomers such as silicone rubber, natural rubber, and butadiene rubber are frequently used. These elastomers have a versatile role in making stretchable devices, such as wearable electronics. Such composites have the potential to revolutionize industries such as aerospace, automotive, electronics, and healthcare. Key aspects covered by this Special Issue include the following:

(1) **Nanofillers**: Nanofillers are the organic or inorganic additives used in polymer matrices to improve their mechanical, electrical, or thermal properties. These additives confer on the final composite robust properties and make them useful for industrial applications as energy harvesters, strain sensors, etc. [3]. Moreover, the morphological features of nanofiller additives are a matter of interest. For example, carbon nanotubes with one-dimensional morphology and a high aspect ratio are helpful for improving electrical conductivity. Furthermore, two-dimensional graphene with a sheet-like morphology is useful for improving barrier properties. Thus, researchers have different options for nanofillers to fulfill the target property of interest.
(2) **Polymer matrix**: Polymer matrices are composed of macromolecules of different types, such as thermoplastics, thermosets, and elastomers. Among them, elastomers are most promising because of their unique properties, such as their ability to stretch under mechanical deformation and bounce into the original shape once the strain is removed [4]. The properties of a polymer matrix strictly depend on the type of polymer matrix used during composite preparation.
(3) **Polymer composites**: Polymer composites are materials that are based on a combination of nanofillers with a polymer matrix. The properties of these polymer composites are strictly based on the type of polymer matrix and nanofiller additives used during fabrication [5]. Moreover, the fabrication procedure used affects the final properties of these composites. The fabrication method could be melting mixing, solution mixing, or in situ polymerization. Among polymer composites, the composites based on elastomers as a polymer matrix are the most promising. This is due to their usefulness in a wide range of applications, such as energy harvesting and automobile tires.
(4) **Concept of new horizons in polymer composites**: Here, “new horizons” refers to the innovative and novel ideas explored by researchers working in the polymer composite field around the world. These novel ideas include the application of cutting-edge findings to obtain new and improved properties, making composites more robust for engineering applications [6]. These developments may include the emergence of a new class of materials, a new type of polymer matrix, or a new manufacturing technique.
(5) **Industrial applications**: Improving polymer composite properties through the selection of new-generation additive materials in a polymer matrix is a hot topic of research. Their use for the development of particular applications is an interesting subject, e.g., tuning stiffness for high-load applications such as tires or soft applications such as tissue engineering [7]. The properties of these polymer composites can be tuned with respect to a specific application. Thus, the subject of this Special Issue is a matter of interest for the readership of the *Polymers* journal.
(6) **Advantages of nanofillers used in polymer composites**: There are various advantages of using nanofillers in polymer composites, such as (a) enhanced properties of the filler additives at the nanoscale, providing uniform dispersion in the polymer matrices; (b) the nano-effect of these additives provides high reinforcement, higher tensile strength, and higher elongation at break compared to traditional fillers like carbon black; (c) nanofillers are typically lightweight and therefore useful for various smart applications, such as wearable electronics; (d) nanofillers exhibit higher electrical and thermal conductivity due to uniform dispersion and thus are useful for higher engineering applications; (e) the high surface area of these nanofillers provides a higher interfacial area for polymer chains to interact with filler additives and leads to improved properties; and (f) nanofillers can mitigate the shrinkage of polymer composites during the fabrication of polymer composites, thereby leading to improved dimensional stability of the final products [8]. Overall, the careful selection and optimization of the nanofiller additives is necessary to obtain properties and applications of interest.
(7) **Challenges in polymer composites**: There are various advantages of using nanofiller additives in polymer matrices for polymer composites. However, there are a few challenges with respect to properties and final engineering applications. For example, monolayer graphene is a great additive as a nanofiller in polymer composites, but synthesizing it at a large scale is difficult and very expensive. Moreover, in energy-harvesting tests, the mechanical stability of electrodes and substrates based on polymer composites is challenging, and limited mechanical stability influences the long-term durability of the final device [8].
(8) **Environmental impact and sustainability**: With the emergence of new technologies in polymer composites to achieve high performance, the carbon footprint of the process and its sustainability are of the utmost importance. The contribution of non-biodegradable polymers like polyethylene to global warming and pollution needs to be considered [9]. The use of sustainable and environmentally friendly, biologically degradable polymers is the focus of researchers globally. Hence, this Special Issue focuses mostly on environmentally friendly polymer composites.

Keeping all these aspects in mind, the present Special Issue presents a collection of articles (Communication, Research Articles, Review Articles) on polymer composites reinforced with nanofillers and their usefulness for different applications. More specifically, the key areas addressed include but are not limited to (a) all types of organic or inorganic nanofillers in the pristine or functionalized state, (b) all types of polymer matrices, (c) all types of properties, multi-scale modeling with theoretical aspects in polymer composites, and (d) finally self-healing, aging, and durability of composites. This Special Issue contains 10 articles from researchers across the globe covering the diverse topic of polymer composites, as summarized below.

## 2. Overview of Published Articles

Ortaç et al. [1] present a novel route of obtaining high thermal conductivity and phase change properties of a polymer nanocomposite. These nanocomposites were filled with boron nitride and doped with lead oxide nanoparticles to achieve enhanced properties. At 13 wt%, the thermal conductivity (λ) of the nanocomposites was 18.874 W/(mK). Moreover, the crystallization fraction (FC) with different co-polymers was 0.032, 0.034, and 0.063. The key takeaway from this study is that the composites present in this study can be used as energy storage materials due to their versatile nature. Kumar et al. [2] present an interesting method of reinforcing silicone rubber with a triple-filler hybrid system (carbon nanotubes, clay minerals, and iron particles) to obtain a robust magneto–mechanical performance. The compressive modulus was 1.73 MPa (control), 3.9 MPa (MWCNT, 3 phr), 2.2 MPa (clay mineral, 2 phr), 3.2 MPa (iron particle, 80 phr), and 4.1 MPa (hybrid system, 80 phr). Moreover, the results show that the triple-filler system emerged as the best candidate due to its high mechanical stiffness, which is useful for high load applications; optimum fracture strain; and tensile strength. Finally, the triple-filler system exhibits efficient magnetic sensitivity and significant output voltage as an energy-harvesting device.

Kistaubayeva et al. [3] investigated the influence of encapsulating a prebiotic with a biological origin polymer system on probiotic survival. The average size of the hybrid symbiotic beads was 3401 µm (wet) and 921 µm (dry). Moreover, the bacterial titer was 10^9^ CFU/g. These results show the promising prospect of encapsulating prebiotics for a delivery system. Kaptan et al. [4] evaluated the surface roughness and flexural and micro-tensile strength of composites based on glass-ionomer-based composites in vitro. Among the different samples, the highest roughness achieved was 0.33 ± 0.1 while the lowest roughness obtained was 0.17 ± 0.04. Similarly, the highest flexural strength obtained was 86.32 ± 15.37, while the lowest one was 41.75 ± 10.05. Moreover, bonding among the materials was noticed between self-cured Cention N and other traditional composites. Zhang et al. [5] conducted a predynamic study on concentrate composites reinforced with polyvinyl alcohol. At a center deflection of 40 mm, the peak force increased from 3700 (PVA content 0.5 wt%) to 4700 (PVA content 2 wt%). These improvements are beneficial for the use of these composites for high-load-bearing applications. Abdel-Gawad et al. [6] fabricated composites based on Nylon 6 and clay minerals and reported their mechanical and structural properties. The results show that the solution mixing method used was more promising for the dispersion of clay minerals than traditional melt mixing. Moreover, the crystallinity of the control sample, which was originally 36%, increased to 58% after the addition of clay minerals in the Nylon-6 matrix. These prospects make these composites useful for high-performance applications.

Cuenca-Bracamonte et al. [7] conducted a study on composites based on a polyetherimide matrix reinforced with reduced graphene oxide or a hybrid of graphene oxide and barium titanate. The results show that the electrical conductivity, using 20 wt% filler, was ~10^−9^ S/cm for the hybrid filler, ~10^−7^ S/cm for rGO, and ~5 × 10^−6^ S/cm for rGO and the hybrid filler combined. Kolev et al. [8] investigated composites based on polycrystalline Y-type hexaferrate and the influence of the magnetic field on the properties of such composites. The highest microwave reflection, 35.4 dB, was achieved at 5.6 GHz without a magnetic field. However, under a magnetic field, it was 21.4 dB at 8.2 GHz. Zare et al. [9] present a power law model and percolation threshold in electrical conductivity for biosensing applications. Their study supports the use of graphene-filled nanocomposites, and the optimized model shows their use as biosensors. Finally, Kumar et al. [10] present an interesting study that deals with the fabrication of composites based on silicone rubber under cyclic and static strain for energy harvesting and magnetic sensitivity. The composites showed promise in their use for energy generation, with an output voltage around 10 volts and a durability of more than 0.5 million cycles. Similarly, the stretchability of the energy-harvesting device was 89% (control), with higher values found for GNP (109%), iron oxide (105%), and the hybrid (133%).

## 3. Summary and Future Outlook of Nanofillers in Polymer Composites

In the last three decades since the discovery of carbon nanotubes in 1991, the demand for nanofillers has increased significantly [10]. Additionally, when comparing them with traditional fillers like carbon black, using carbon nanotubes as nanofillers results in better properties at 3-5 phr compared to carbon black at a 60 phr loading. Thus, carbon nanotubes emerge as a promising additive for polymer composites. The addition of carbon nanotubes as an additive in a polymer matrix improves all properties except the barrier properties. Moreover, graphene emerged as a nanofiller due to its tremendous potential to improve the properties of polymer composites [11]. Moreover, the use of graphene as an additive also improves the barrier properties, which makes it advantageous over carbon nanotubes [8,10]. Thus, the increasing demand for such nanofillers has a tremendous impact on polymer matrices, especially as a reinforcing filler. Moreover, the influence of nanofillers is not only limited to their reinforcing effect; it also makes them suitable for various industrial applications as strain sensors and energy harvesters [8,12]. Keeping these aspects in mind, this Special Issue analyzes the potential impacts of using nanofillers for polymer composites and supports a promising future for materials scientists globally. Overall, the contributions of researchers to this Special Issue present a good quality of research work covering all possible topics within the scope of this field. Key aspects to consider in future research include the following:

(1) **Novel and robust nanofillers as additives**: As supported by the literature and the articles covered in this Special Issue, nanofillers show unique properties and multifunctionality. This aspect allows researchers to tailor the composites with respect to properties and applications of interest. Thus, nanofillers hold promise for scientists working in the polymer composite field.
(2) **Advanced processing and manufacturing**: The continuous efforts of scientists in improving manufacturing techniques have led to better filler dispersion. Additionally, other aspects related to the fabrication of polymer composite will continue to evolve. This will help in optimizing properties and indicates the promising multifunctionality of such composites.
(3) **Medical compatibility**: The use of nanofiller-reinforced polymer composites also indicates a promising future in the nanomedicine field. For example, polymer composites with nanofiller additives with improved biocompatibility and functionality have promising applications in drug delivery and medical implants.
(4) **Eco-friendly and green polymer composites**: With the advances in polymer composite science, the emergence of bio-based nanofillers as additives for polymer composites holds promise. These so-called green polymers are not only environmentally friendly materials but also possess high performance and multifunctionality.
(5) **Promising technologies and integrated performance**: Polymer composites reinforced by nanofillers could find use in new-generation applications such as self-powered electronic devices, flexible and stretchable electronic devices, advanced energy storage, and lightweight, cost-effective additive materials.

Overall, the use of nanofillers as additives represents a subject of interest in the polymer composite field. With continuous efforts from scientists, this field is expected to grow further, providing new cutting-edge technologies for multifunctional applications. Finally, the articles presented in this Special Issue provide insight into new advances and routes for further research and development in this area.
